# Tuning Lignite Structure via Hydromodification To Promote the Formation of Coal-Based CNTs: Exploration for the Carbon Source of CNTs

**DOI:** 10.1021/acsomega.3c01736

**Published:** 2023-07-13

**Authors:** Qingxiang Guo, Yuqiong Zhao, Yaning Lei, Guoqiang Li, Yajun He, Guojie Zhang, Yongfa Zhang, Kunjie Li

**Affiliations:** † State Key Laboratory of Clean and Efficient Coal Utilization, Taiyuan University of Technology, Taiyuan 030024, Shanxi, China; ‡ Key Laboratory of Coal Science and Technology, Ministry of Education, Taiyuan University of Technology, Taiyuan 030024, Shanxi, China; § Shanxi Huaxin Gas Energy Research Institute Co., Ltd., Taiyuan 030032, Shanxi, China

## Abstract

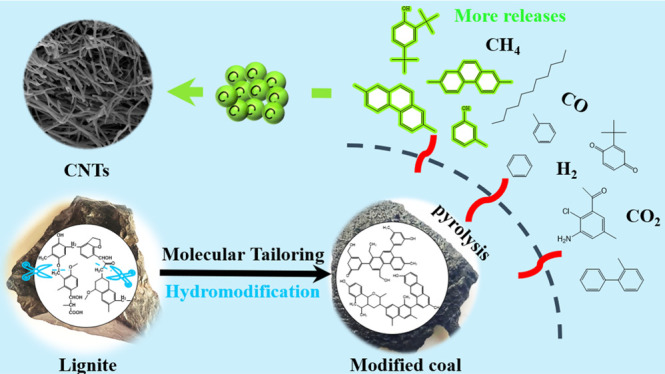

Although the preparation of coal-based carbon nanotubes (CNTs) has been realized in many studies, the relationship between carbon source structure of coal and CNT growth has not been studied in depth. In this study, we used lignite and KOH as raw material and catalyst and tuned lignite structure via hydrothermal modification to promote the formation of CNTs during catalytic pyrolysis. The main carbon source of CNTs was explored from the change of coal structure and pyrolysis characteristics. The results indicate that the CNT yield of lignite pyrolysis products is only 2.39%, but the CNT yield increases significantly after lignite was hydrothermally modified in a subcritical water–CO system. The graphitization degree, the order degree, and CNT content increase continuously with the increase in modification temperature, and C-M_340_ has the highest CNT content of 9.41%. Hydromodification promotes the rearrangement of aromatic carbon structures to generate more condensed aromatic rings linked by short aliphatic chains and aromatic ether bonds. The variation of these structures correlates well with the formation of CNTs and leads to the change in the carbon source components released during coal pyrolysis. Compared to lignite, modified coal releases more aromatic compounds, especially polycyclic aromatic hydrocarbons with ≥3 rings and phenols during catalytic pyrolysis, which is conducive to the transformation into carbon clusters and provides carbon sources for CNT growth. In addition, modified coal releases a slightly more carbon-containing gas (CH_4_ and CO) than lignite, which has a limited effect on the growth of CNTs. This study provides a novel and efficient method for enhancing the growth of CNTs by a molecular tailoring strategy of coal.

## 1 Introduction

Coal is one of the plentiful carbon sources with a three-dimensional (3D) cross-linked network, which is composed of an aromatic/hydroaromatic nucleus linked by bridge bonds.^1,2^ The abundant reserves and distinct structural features of coal make it attractive as cost-effective resources to prepare carbon materials. In recent years, various coal-based carbon materials with diverse structures and properties have been constructed.^3−6^ As one of the most important one-dimensional carbon materials, carbon nanotubes (CNTs) exhibit unique properties such as large specific surface area, high physicochemical stability, and exceptional electrical conductivity, which endow them with great potential to use as key components in carbon composites.^7−10^ Thus far, fabrication of CNT composites derived from coal and its derivatives has caught much attention in terms of adsorption, energy storage, and enhancing the mechanical properties.

Hao et al.^11^ prepared porous carbon/CNT composites by introducing pre-synthesized CNTs to porous carbon. The material with added CNTs can retain the 3D honeycomb-like hierarchical porous structure during thermal treatment. Compared with direct addition of pre-synthesized CNTs to carbon composites, the in situ CNT formation strategy would reduce the overall production cost of CNT-containing composites. Song et al.^12^ developed a method for in situ preparation of multiwalled CNTs (MWCNTs) through Co-catalyzed pyrolysis of coal tar pitch. Large amounts of MWCNTs were formed at 1173 K and the in situ formation of MWCNTs led to evident improvement in the mechanical strength of refractory. Yuan et al.^13^ fabricated N, O co-doped CNTs and activated carbon composites by co-pyrolysis of KOH and urea with bituminous coal. The in situ growth of CNTs provides an open nanoscale scaffold for the carbon composites and promotes the formation of multi-layered macro-meso-microporous structure, which enhances its CO_2_ absorption capacity. The results mentioned above have evidenced the potential applications of CNT composites prepared via catalytic pyrolysis of coal and its derivatives.

In fact, catalytic pyrolysis of coal might be an alternative procedure for large-scale production of CNT-containing composites at lower cost instead of the drastic methods available.^14^ Thus, the effects of catalyst and pyrolysis conditions on the in situ growth of CNTs in coal-based carbon composites have been widely explored. Das et al.^15^ reported that weak crosslinks of coal broken and reactive hydrocarbons were released during the co-pyrolysis of coal and a mixture of KOH and NaOH, such as alkynes and aromatics, which are involved in the formation of carbon nanomaterials. Zhang et al.^16^ fabricated CNTs by potassium-catalyzed pyrolysis of bituminous coal, and the CNTs in pyrolysis products have a good graphite crystal structure. By adding iron to increase the heat transfer rate, Reddy et al.^17^ prepared good quality CNTs and nanoparticles in the heat-treated coal char, and the diameters of carbon nanostructures increased with the agglomeration of Fe particles. Lv et al.^18,19^ successfully synthesized CNTs by the Fe-K catalytic pyrolysis of bituminous coal at 900 °C. K etches the amorphous carbon atoms of coal and C atoms dissolved on the Fe atom surface to form a Fe-C solid solution, leading to the growth of large numbers of CNTs. However, the further increase of CNTs content in carbon composites is limited because the hydrocarbon molecules released during catalytic pyrolysis are closely related to the inherent structure of coal. Hence, it is crucial to tune the structure of coal to active precursors for constructing CNT composites via molecular tailoring strategies.^20^

Hydromodification of coal in a subcritical-CO system is an important method for tuning the structure and property of coal.^21^ In this system, the original cross-links of coal would be broken and a large quantity of active hydrogen is generated by water gas shift reaction, which improves the mobility of radical fragments and promotes the reconstruction of molecules.^22−25^ It is anticipated that this system could be employed to tailor the coal structure into active precursors. However, little research has so far been dedicated to explore whether and how the hydromodification of coal affects the growth of CNTs in carbon composites.

In this work, the impact of coal hydromodification on the formation of CNTs during catalytic pyrolysis was investigated. Special attention was paid to find the interrelation between the coal structural changes and the growth of CNTs. The evolution of pyrolysis products has also been studied to explore the carbon source of CNTs. This research develops an effective strategy to improve the formation of CNTs in carbon composites and provides a new perspective for high-added value utilization of lignite.

## 2 Results and Discussion

### 2.1 Effect of Hydromodification on the Growth of CNTs

**Figure 1:**
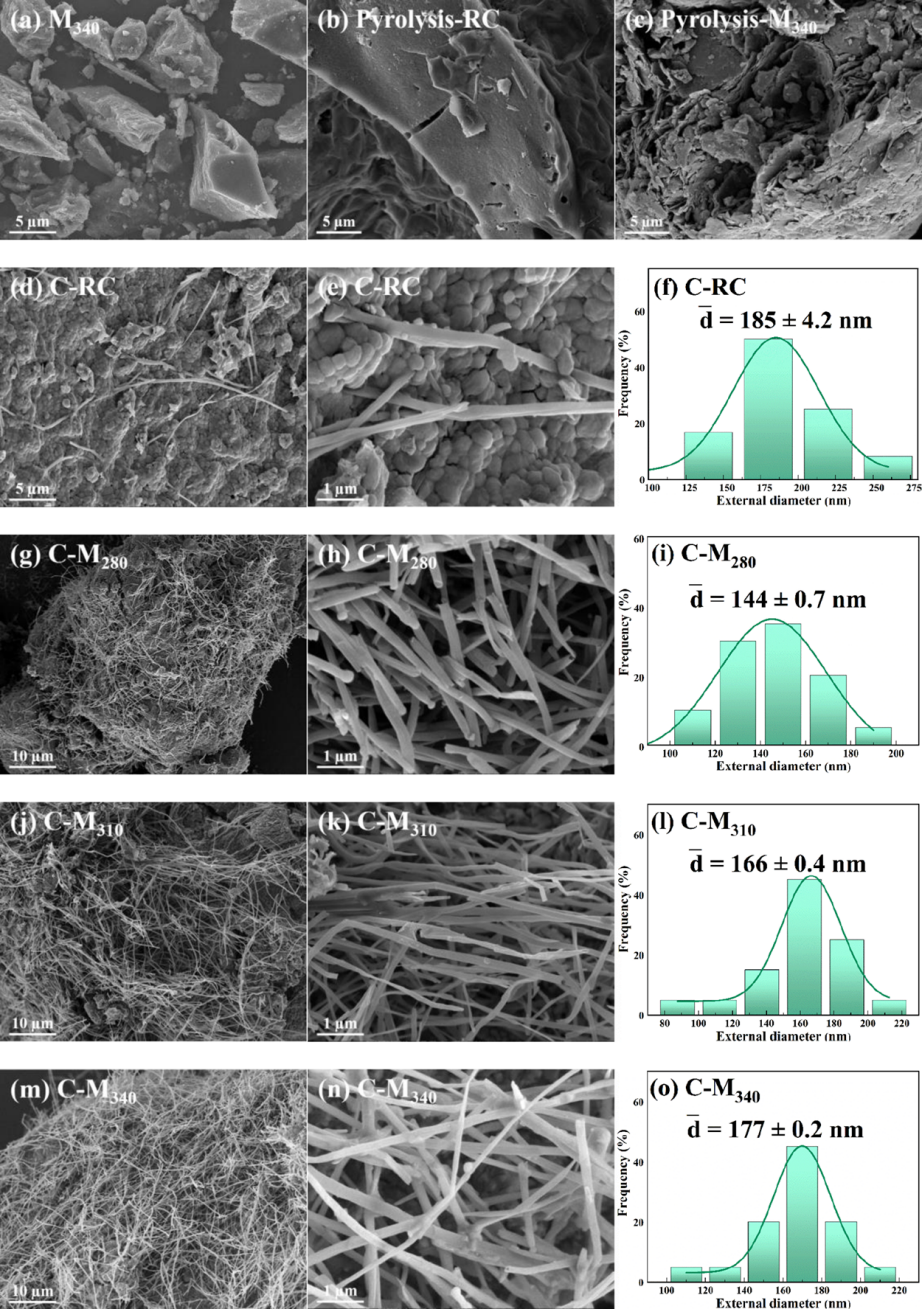
SEM images and outer diameters of CNTs: (a) M_340_, (b) pyrolysis-RC, (c) pyrolysis-M_340_, (d–f) C-RC, (g–i) C-M_280_, (j–l) C-M_310_, and (m–o) C-M_340_.

Figure 1a shows the scanning electron microscopy (SEM) images of modified coal M_340_; no CNTs were observed, which indicates that the role of hydrothermal modification is to change the structure of lignite, but not to form CNTs directly. Figure 1b,c shows the pyrolysis products of raw coal and modified coal M_340_ without KOH, which are mainly smooth coal char, and no CNTs are generated. However, under the action of KOH, CNTs were observed in the pyrolysis products of raw coal and modified coal (Figure 1d,m), which indicates that KOH has the catalytic effect on CNT growth. Zhang et al.^16^ believed that the K catalyst has dual functions of etching the carbon skeleton of coal to generate carbon source and catalyzing the formation of CNTs. Metal K can convert aromatic carbon and ether carbon structure into carbon atoms or carbon clusters and then into CNTs.

As shown in Figure 1d,g,j,m, only a small amount of CNTs was scattered on the coal char surface in C-RC. However, the apparent content of CNTs in pyrolysis products increased significantly after the hydromodification of lignite in a subcritical water–CO system, especially, in C-M_340_. Moreover, the average outer diameter of CNTs increased gradually from ca.144 to 177 nm with the increase in the modification temperature (Figure 1i,l,o). This phenomenon indicates that the change of coal structure has an important influence on the quantity and structure of CNTs; the hydromodification in a subcritical water–CO system can effectively improve the carbon structure of lignite and then promote the generation of CNTs during catalytic pyrolysis.

**Figure 2:**
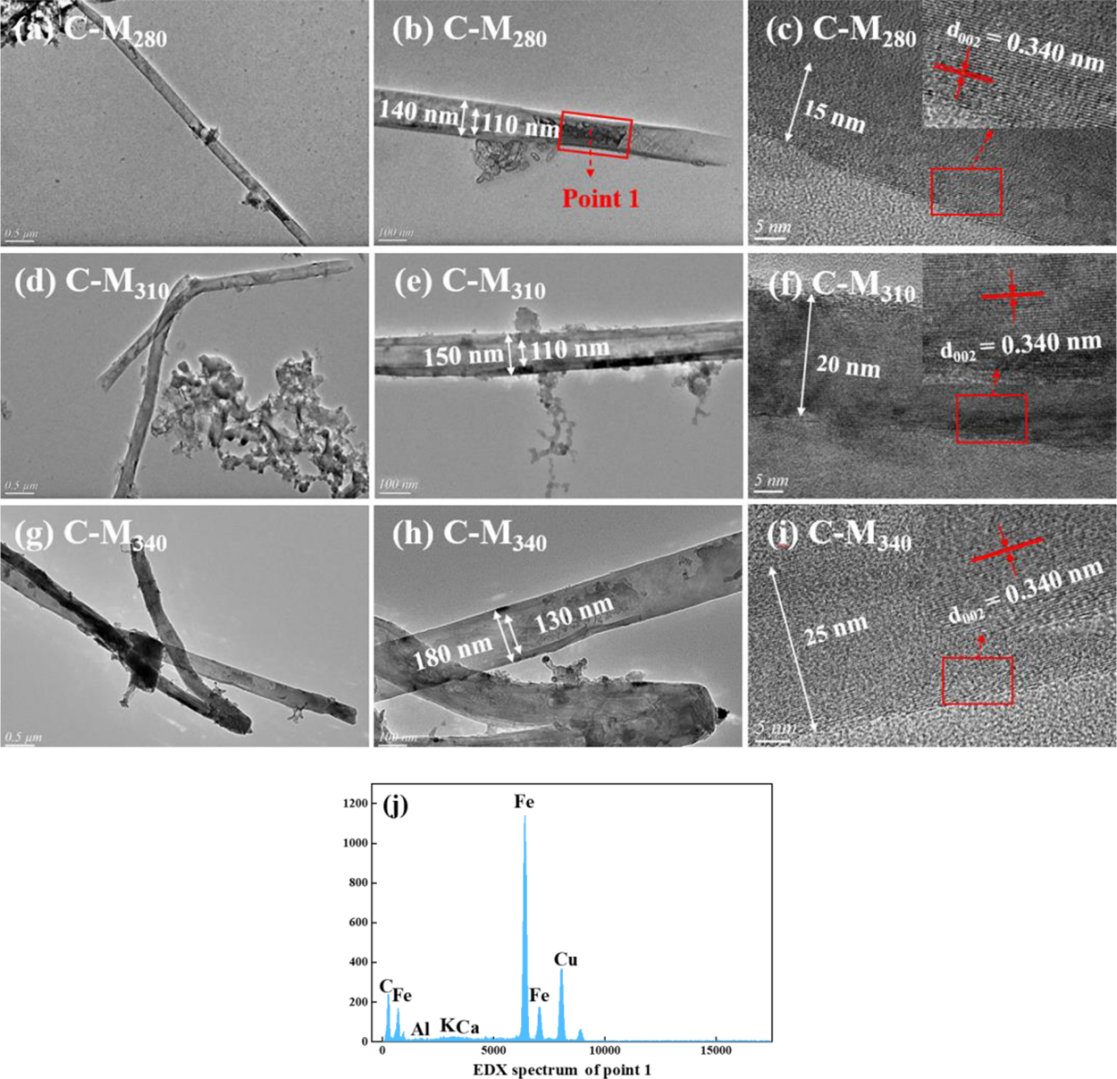
TEM images of CNTs: (a–c) C-M_280_, (d–f) C-M_310_, (g–i) C-M_340_, and (j) EDX spectrum of point 1.

As shown in Figure 2, the formed CNTs were mainly multi-walled CNTs with uniform diameters. The outer diameters of the as-prepared CNTs ranged from 140 to 180 nm, and their inner diameters ranged from 110 to 130 nm. The interlayer spacing of graphite (Figure 2c) was about 0.340 nm, which was attributed to the (002) plane of graphite. The tube walls of CNTs were composed of about 40–70 layers of graphene. Furthermore, agglomerated Fe metal was found in the center of the CNTs (Figure 2b), which indicated that the iron minerals in coal were involved in the formation of CNTs.^30^ It is generally believed that the carbon source produced by coal decomposition undergoes the process of “dissolution-diffusion-precipitation″ on the Fe catalyst surface, forming a large number of CNTs.^31,32^ The Fe particles were located in the middle of the tube instead of the ends, indicating that the Fe particles were in a molten state at high temperature and migrated during the growth of CNTs.^33,34^

**Figure 3:**
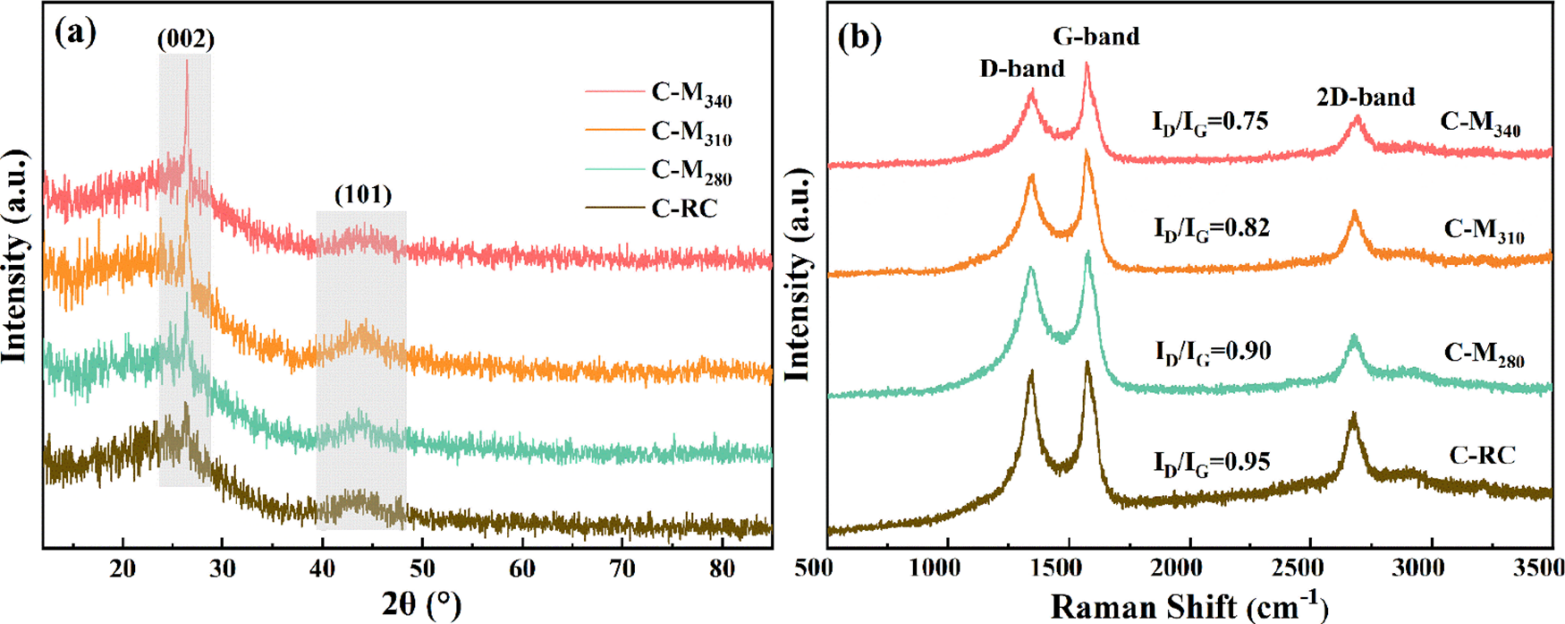
XRD (a) and Raman (b) patterns of coal pyrolysis products.

Figure 3a shows the X-ray diffractometry (XRD) patterns of C-RC and C-Mt. Sharp diffraction peaks were observed at 2θ = 26.4°, corresponding to the characteristic (002) peaks of graphite (PDF#41-1487). The (002) peak intensity of each sample decreased in the order of C-M_340_ > C-M_310_ > C-M_280_ > C-RC. The (002) peak intensity was enhanced with the increase in the modification temperature, which indicates that the graphite carbon structure is increasing. In order to further confirm the corresponding relationship between the CNT content in composites and the peak intensity of 002, C-M_340_ was oxidized at 550 °C. Based on the fact that the excellent graphitization of CNTs endows its better thermal stability than amorphous carbon, almost only CNTs remained in the sample after the oxidation treatment for 30 min (Figure S1). Figure S2 shows the XRD patterns of the C-M_340_ samples after oxidation treatment at 550 °C for different times, and the intensity of (002) peak increased continuously with the removal of amorphous carbon and the increase of CNT content. Therefore, the increase of the (002) peak intensity reflects the generation of a large number of CNTs to some extent.

The order degree and internal defects of carbon materials can be further analyzed by Raman spectra.^12^Figure 3b shows Raman spectra of C-RC and C-Mt. Among the peaks observed, the D-band at 1350 cm^–1^ is caused by defects, vacancies, and amorphous carbon impurities. The G-band at 1590 cm^–1^ associated with the splitting of the E2g stretching mode for graphite. The 2D band at 2700 cm^–1^ corresponded to the second order of the D-band, which is related to the graphene layer number.^35^ Generally, the order degree of CNTs is expressed by the peak intensity ratio of the D-band and G-band (*I*_D_/*I*_G_). A lower ratio indicates fewer internal defects of CNTs. The *I*_D_/*I*_G_ of C-RC was 0.95, indicative of a low degree of graphitization of C-RC. In case of the C-Mt, *I*_D_/*I*_G_ decreased from 0.90 to 0.75 with the increase in the modification temperature, suggesting that the order degree of the carbon structure constantly increases. This phenomenon indicates that the increase in the modification temperature is beneficial to the generation of more ordered carbon structures during catalytic pyrolysis.

**Figure 4:**
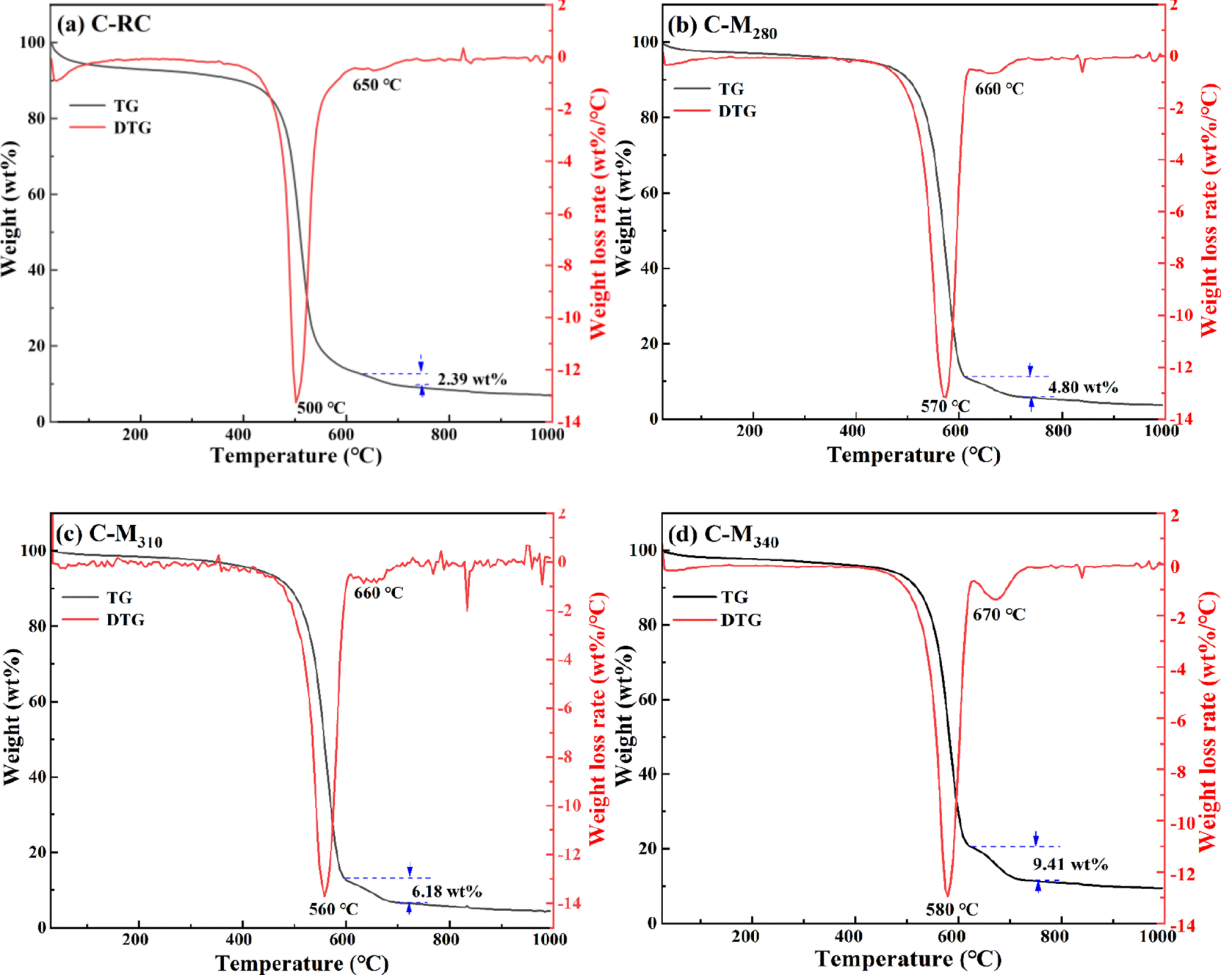
TG-differential TG analysis of coal pyrolysis products: (a) C-RC, (b) C-M_280_, (c) C-M_310_, and (d) C-M_340_.

**Figure 5:**
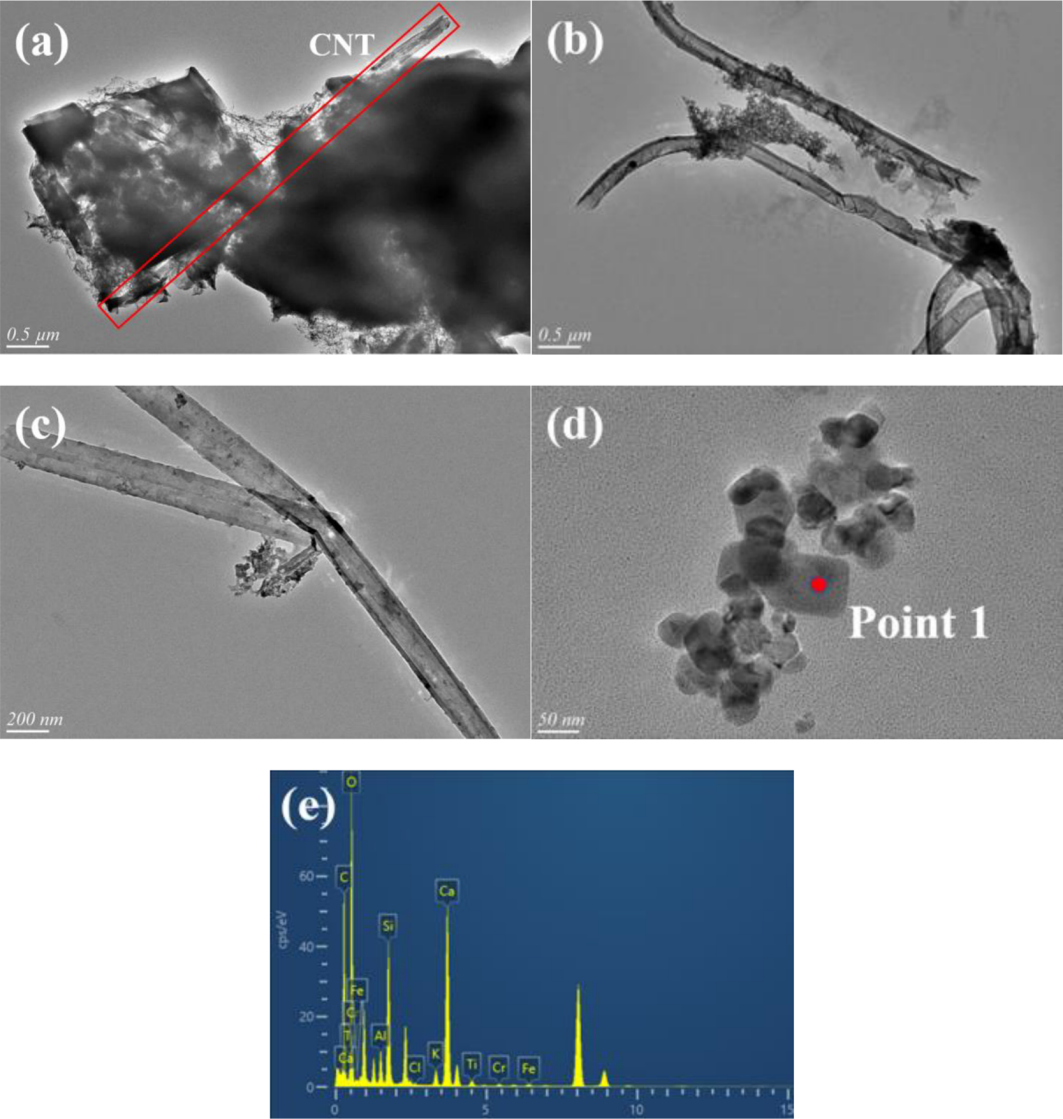
TEM images of C-M_340_ after oxidation at different temperatures: (a) 550 °C, (b) 580 °C, (c) 670 °C, and (d) 700 °C; (e) EDX spectrum of point 1.

Thermogravimetric analysis (TGA) can be employed to characterize the thermal stability of carbon nanomaterials and the content of different carbon types.^36^Figure 4 shows the TG-differential TG curves of coal pyrolysis products. Obviously, the larger and smaller loss peaks were observed in 500–580 and 650–670 °C. In order to distinguish the carbon forms represented by these two peaks, C-M_340_ was heated to different temperatures for oxidation treatment. When C-M_340_ was heated to 550 °C for oxidation treatment, the CNTs were covered with amorphous carbon (Figure 5a). When the oxidation temperature rose to 580 °C, the amorphous carbon around CNTs has been significantly reduced. This phenomenon indicates that the thermal stability of the CNTs is better than that of the amorphous carbon, and the mass reduction of the first weight loss peak is mainly for the amorphous carbon. Furthermore, when C-M_340_ was heated to 670 °C, almost no amorphous carbon was observed around the CNTs (Figure 5c). When the temperature was raised to 700 °C, only ash and no CNTs were observed (Figure 5d,e). This indicates that the mass reduction of the second weight loss peak is mainly for the CNTs.^37^

Therefore, the larger the second weight loss peak, the larger the CNT content of the samples. The second weight loss peak of C-RC accounted for ∼2.39%, which was the smallest (Figure 4a). The proportions of the second weight loss peaks of C-M_280_, C-M_310_, and C-M_340_ were 4.80, 6.18, and 9.41%, respectively. This phenomenon shows that with the increase in the modification temperature, the CNT content in the composites increased significantly. When the modification temperature continues to increase, the CNT content in composites still tends to increase (Figures S3 and S4). In addition, the two weight loss peaks of C-RC were observed at 500 and 600 °C, respectively, while corresponding peaks of the modified coal pyrolysis products were observed at 560–580 and 660–670 °C, and the weight loss peaks tended to move to a higher temperature range with increasing modification temperature. This result suggests that the increase of CNT yield is beneficial to improve the overall thermal stability of material. Compared with similar research (Table 1), this study provides a novel and efficient method for enhancing the growth of CNTs by adjusting the lignite structure, and C-M_340_ exhibits the highest degree of graphitization and CNT content.

**Table 1:** CNT Yield between This Work and the Related Literature

raw material	catalyst dosage	CNT yield (wt %)	source
bituminous coal	C:KOH = 2:1	10.16	(41)
bituminous coal	C:K_2_CO_3_ = 1:1	4.08	(19)
bituminous coal	C:K_2_CO_3_:Fe(C_5_H_5_)_2_ = 2:1:1	17.88	(19)
coal tar pitch	C:Co(NO_3_)_2_ = 1:0.75	40.00	(12)
lignite	C:KOH = 1:1	2.39	this work
modified coal	C:KOH = 1:1	9.41	

### 2.2 Effect of Hydromodification on the Coal Structure

#### 2.2.1 XRD Analysis

**Figure 6:**
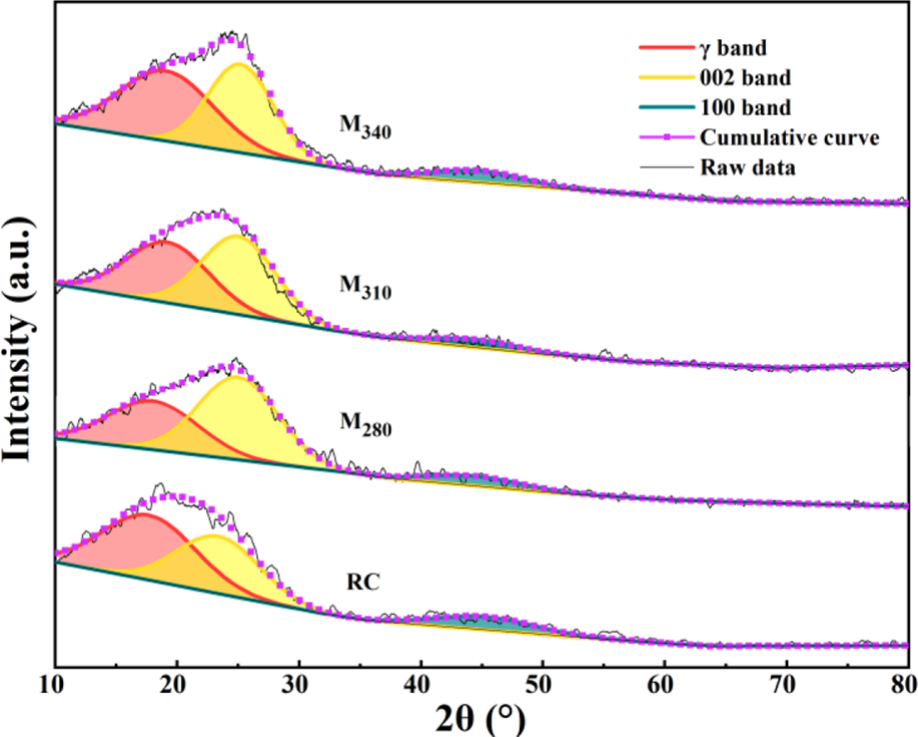
Curve-fitting of XRD patterns of coal samples.

To investigate the effect of lignite structure changes on the preparation of CNTs by catalytic pyrolysis, the crystal structures of lignite and modified coals were analyzed by XRD. Figure 6 shows the three Gaussian peak fits for the 18, 25, and 44° bands. The (002) band at 25° reflected the stacking height of the aromatic layers.^38^ The γ band on the left of the (002) band corresponded to the aliphatic hydrocarbon and alicyclic structures connected to the aromatic rings in coal.^27^ The (100) band at 44° corresponded to the diameter of the aromatic ring structure. It can be observed from Figure 6 that the intensity of the (002) band of modified coal is higher than that of RC, and the intensity gradually increased with the increase in the modification temperature, indicating that hydromodification promotes the formation of ordered aromatic structures. The large proportion of the γ band in RC corresponded to the rich aliphatic structure in lignite. However, the area of the γ band in M_280_ decreased significantly, and with the increase in the modification temperature, the area gradually increased (Table 2). This result was caused by the simultaneous decomposition and hydrogenation of coal during hydromodification.^23^ At a low modification temperature, the decomposition of coal was dominant, and the abundant bridge bonds and alkyl side-chain structures in coal were broken, thereby reducing the aliphatic structure. However, with the increase in the modification temperature, the water–gas shift reaction was promoted and a large amount of active hydrogen were generated, thereby enhancing hydrogenation and stabilizing more hydrocarbon radicals, leading to the increase in the aliphatic structure.^23,25^ SEM analysis results reveal that the CNT content of C-M_280_ is higher than that of C-RC, while the content of aliphatic carbon in M_280_ is significantly less than that of RC; hence, aliphatic carbon may not be the main carbon source precursors for CNT generation.

**Table 2:** XRD Structural Parameters of Coal Samples

samples	Aγ/%	2θ_002_/°	*d*_002_/nm	*L*_c_/nm	*L*_a_/nm	*N*
RC	49.63	23.38	0.380	1.06	1.85	3.79
M_280_	36.38	25.00	0.356	1.10	1.92	4.10
M_310_	43.48	25.04	0.355	1.19	2.01	4.34
M_340_	49.04	25.20	0.353	1.36	2.05	4.85

Table 2 summarizes the crystal structure parameters obtained by deconvolution. Obviously, hydromodification causes the (002) band shift from 23.38 to 25.20°, which was closer to the standard (002) peak of graphite (26.6°). Moreover, d_002_ of M_280_ was clearly less than that of RC, and it decreased slightly with the increase in the modification temperature. Simultaneously, *L*_c_ and *N* of modified coals increased gradually, indicative of a more ordered directional arrangement of the aromatic structure in modified coal. This result is related to the fact that hydromodification would destroy the cross-linked structure of coal and render stronger fluidity to the aromatic ring fragments, which is beneficial for the further stacking and rearrangement of aromatic layers.^22,39^ For *L*_a_, the *L*_a_ of modified coal increased with the increase in the modification temperature, indicating that the hydromodification promotes the polycondensation of aromatic carbon. Meanwhile, the CNT content of the coal pyrolysis products increased gradually. Therefore, the aromatic carbon structure of coal is thought to be closely related to CNT growth.

#### 2.2.2 Fourier Transform Infrared Analysis

**Figure 7:**
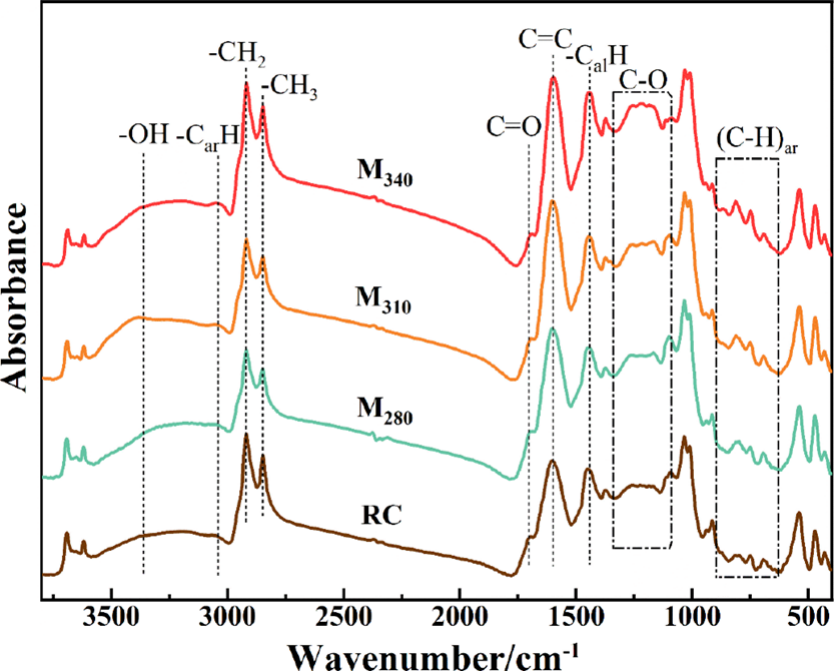
FTIR spectra of the coal samples.

The Fourier transform infrared (FTIR) spectra of lignite and modified coals are shown in Figure 7, which are mainly composed of absorption peaks of aromatic nucleus, aliphatic side chains, and oxygen-containing groups. According to Figure 7, the position of each absorption peak did not shift before and after modification, but the peak intensity changed significantly, reflecting that hydromodification changes the organic macromolecular structure of lignite. Peaks observed at 3060 and 1600 cm^–1^ corresponded to the stretching vibrations of aromatic C–H and C=C bonds, respectively, which increased with the increase in the modification temperature.^29^ This result indicates that hydromodification promotes aromatization and generates an increasing amount of aromatic nucleus (C=C) structures or condensed aromatic rings. This result was also confirmed by the increase in the ratio of aromatic hydrogen to aliphatic hydrogen (H_ar_/H_al_) (Table 3).^28^ Peaks at 1100–1300 cm^–1^ corresponding to the ether bonds and phenolic hydroxyl groups increased after hydromodification, possibly related to the reaction between CO and the coal organic structure.^40^ The peak at 1700 cm^–1^ is caused by the conjugated C=O bond, and the peak intensity decreased; hence, the weak carboxyl group in coal is removed. Furthermore, the C–O/C=O content increased after hydromodification (Table 3), indicating that hydromodification promotes the conversion of carboxyl and carbonyl groups to the ether bond. Zhang et al.^41^ have believed that the carbon source required for CNT growth mainly comes from the aromatic carbon C–C, C–H, and ether carbon C–O–C structures, which would accumulate pure carbon atoms or carbon clusters for the growth of CNTs. In this study, hydromodification promoted the increase in the aromatic carbon and ether bond structures in lignite, increasing a large amount of carbon source precursors for CNT growth.

**Table 3:** FTIR Spectral Parameters of Coal Samples

samples	C–O/C=O	(C–H)_ar_	C_al_	H_ar_/H_al_	CH_2_/CH_3_
RC	3.21	0.7	6.28	0.08	4.25
M_280_	4.08	0.88	4.91	0.09	4.11
M_310_	5.09	1.03	5.48	0.10	3.01
M_340_	6.02	1.07	5.63	0.10	2.54

**Figure 8:**
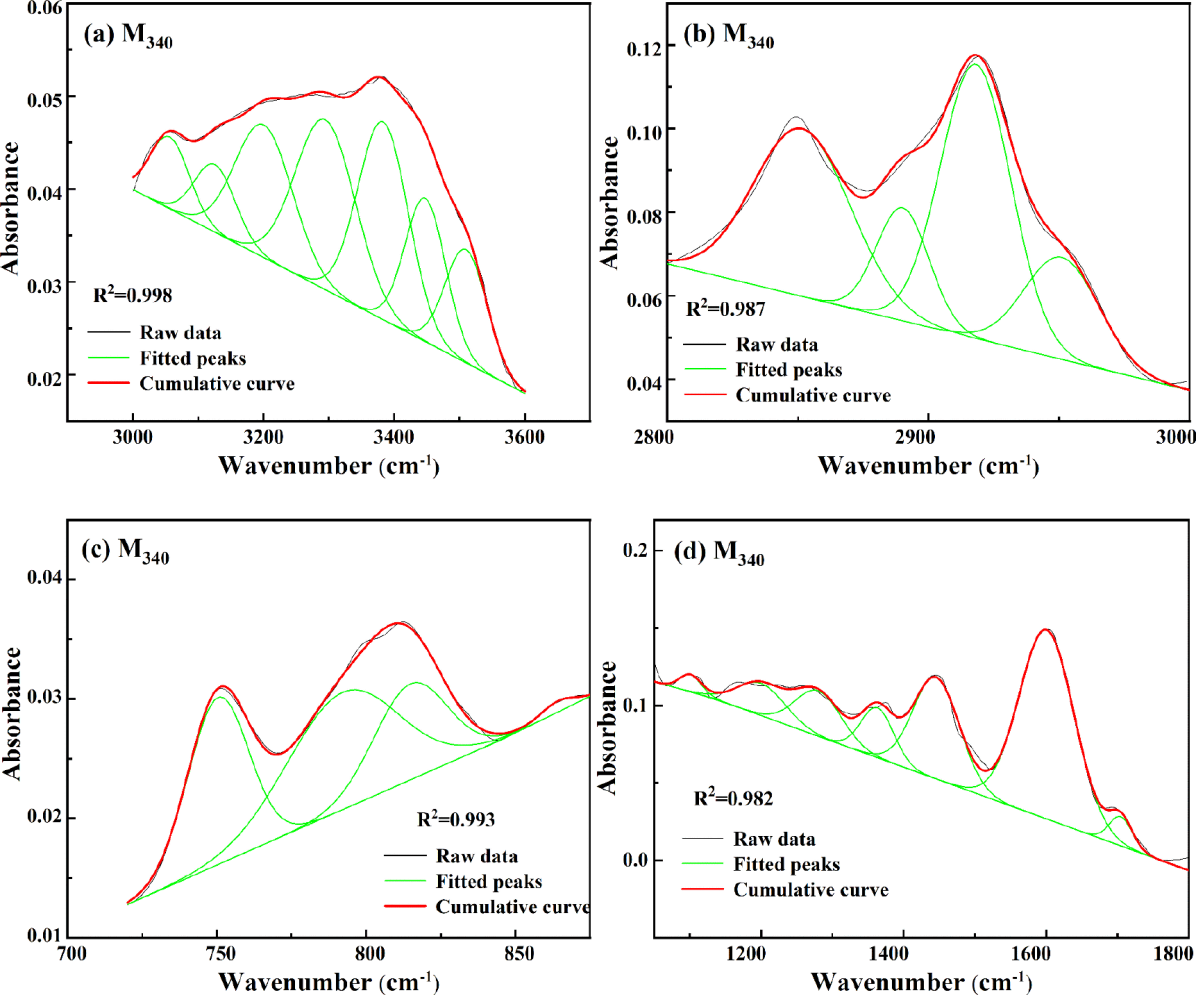
Curve fitting of FTIR spectra of M_340_: (a) 3000–3600 cm^–1^, (b) 2800–3000 cm^–1^, (c) 700–900 cm^–1^, and (d) 1100–1800 cm^–1^.

FTIR spectra of coal samples are fitted with Gaussian curves. Figure 8 shows the curve fitting of M_340_. In addition, Figure S5 shows the fitting results of the other coal samples. Table S1 summarizes the absorption peak areas of different functional groups, and Table 3 lists the FTIR spectral parameters. The content of aliphatic hydrocarbon structures (C_al_) first decreased and then increased, which was consistent with the XRD fitting result, showing that the weak aliphatic chains in coal are first broken and then increase during hydromodification. Moreover, the length of aliphatic side chains (CH_2_/CH_3_) significantly reduced, and (C–H)_ar_ corresponding to the substituted aromatic hydrocarbon content gradually increased, further revealing that the connection form of aliphatic side chains in the coal macromolecular structure is changed during hydromodification. The length of the aliphatic chains was shortened, and the degree of branching was increased, with many short aliphatic chains attached to the condensed aromatic rings being obtained. Based on the XRD and FTIR analysis results, hydromodification is thought to promote the generation of condensed aromatic rings with short aliphatic chains, which is beneficial for the generation of CNTs.

### 2.3 Effect of Hydromodification on the Coal Pyrolysis Characteristics during the Preparation of CNTs

#### 2.3.1 Pyrolysis Product Distribution

**Figure 9:**
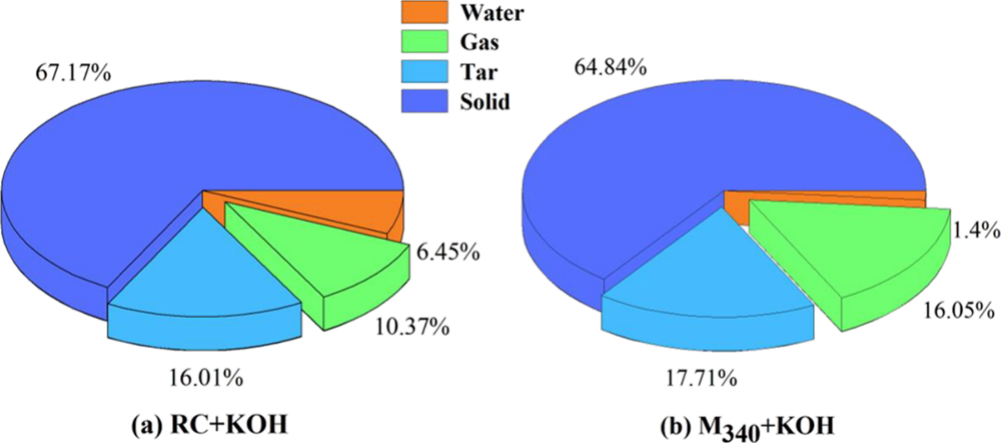
Product distribution of coal pyrolysis catalyzed by KOH: (a) RC and (b) M_340_.

**Figure 10:**
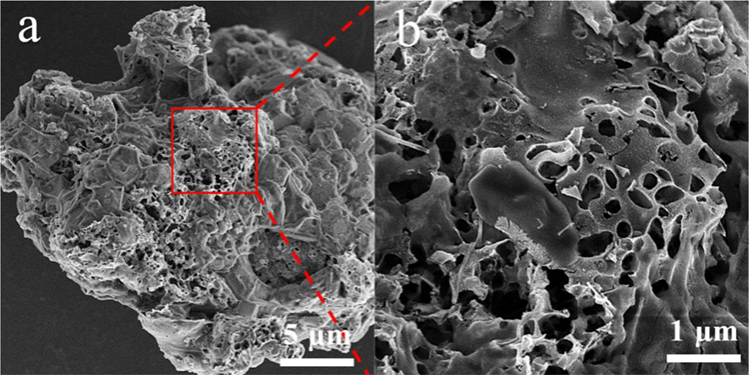
SEM images of the pyrolysis solid product of M_340_: (a) 5 μm and (b) 1 μm.

CNTs are generated during the catalytic pyrolysis of coal. The gas, tar, and char produced by coal cracking were in a sub-stable state at high temperatures, possibly reacting with the K catalyst to release carbon atoms required for CNT growth.^14^ To analyze the components that were more favorable for the growth of CNTs during coal pyrolysis, the pyrolysis characteristics of RC and M_340_ were investigated. Figure 9 shows the yields of pyrolysis products, including water, gas, tar, and solid. The solid product refers to the mixture of char and K compounds. The solid yield of M_340_ decreased by 2.33% compared with that of RC, while the total yields of volatile gas and tar of M_340_ increased by 7.38% compared with that of RC (Figure 9). Among volatile, the tar yield of M_340_ was only 1.70% greater than that of RC, and the gas yield of M_340_ was significantly greater than that of RC by 5.68%. This phenomenon suggests that the cracking of the coal matrix of M_340_ is easier and that metastable components such as gas and tar are released. Notably, after the gas and tar escaped from the coal matrix, the CNTs in the solid product of M_340_ were significantly reduced (Figure 10), indicating that metastable carbon in gas and tar is critical for the growth of CNTs, and the stable carbon in char cannot provide a large amount of active carbon source for the CNT growth. Therefore, hydromodification promotes the release of metastable hydrocarbon, which can provide a large active carbon source for CNT growth.

#### 2.3.2 Pyrolysis Gas Analysis

**Figure 11:**
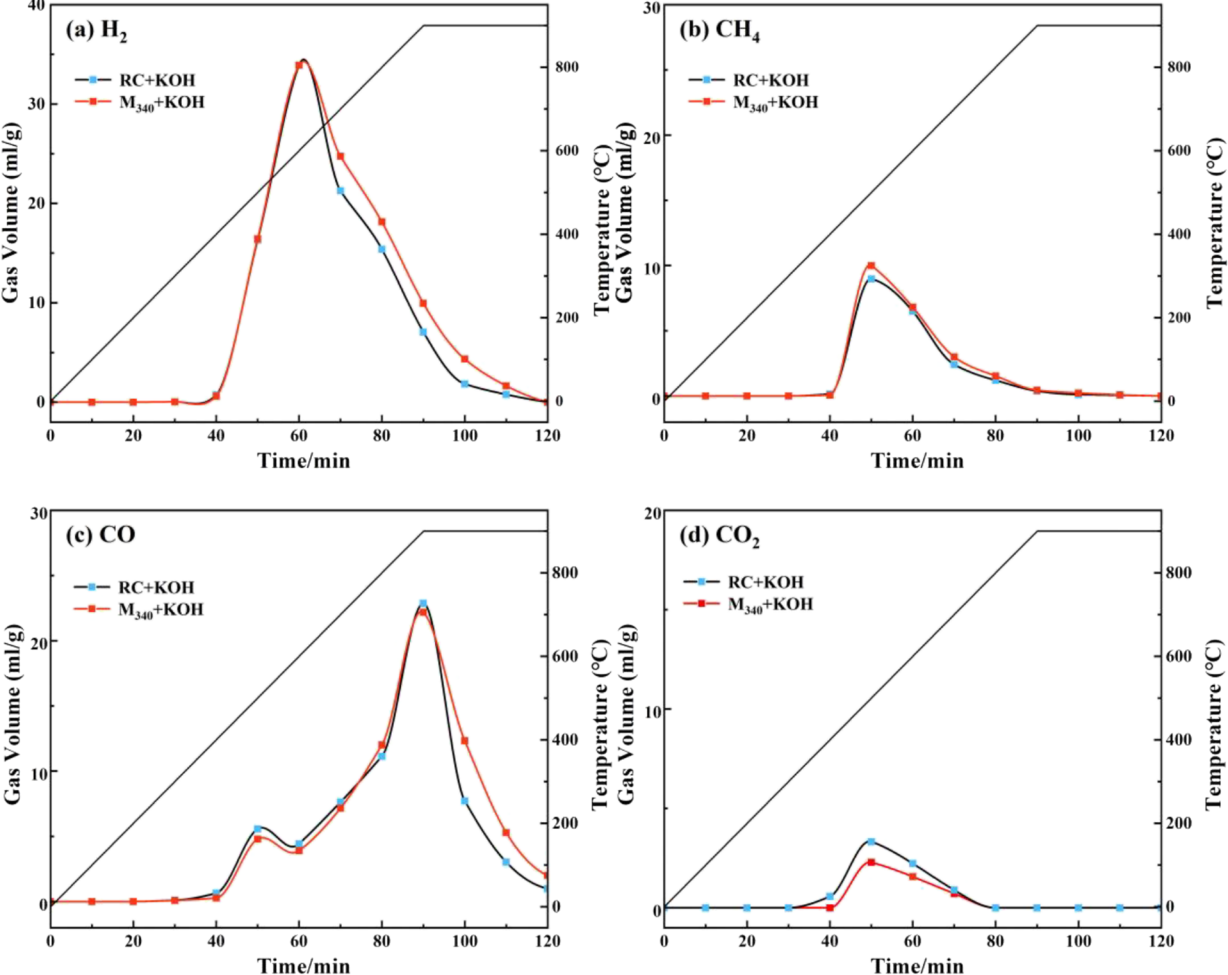
Pyrolysis gas evolution of RC and M_340_: (a) H_2_, (b) CH_4_, (c) CO, and (d) CO_2_.

Figure 11 shows the evolution of the main pyrolysis gases of RC and M_340_, including H_2_, CH_4_, CO, and CO_2_. Generally, H_2_ originates from the cleavage of aliphatic chains, aromatic side chains, and polycondensation of aromatic hydrocarbons.^42^ The H_2_ evolution curves of RC and M_340_ were the same before the temperature reached 600 °C (Figure 11a) because of the similar content of aliphatic hydrocarbons in RC and M_340_. However, the amount of H_2_ released by M_340_ was greater than that released by RC at a temperature greater than 600 °C, indicating that M_340_ undergoes intense polycondensation at high temperatures. Generally, CH_4_ comes from the cleavage of aliphatic hydrocarbons and methoxy groups.^43^ Although the aliphatic hydrocarbon content of RC and M_340_ was similar, the aliphatic side chains of M_340_ were shorter than those of RC, promoting the pyrolysis of M_340_ to generate an increased number of methyl radicals; hence, the CH_4_ yield of M_340_ increases slightly.

There are two different pathways to generate CO during the pyrolysis of coal. The first pathway involved the decomposition of oxygen-containing structures such as carbonyl and ether bonds.^42^ The weak oxygen functional groups in lignite were partially removed during hydromodification,^44^ so the CO release of M_340_ is less than that of RC at ∼500 °C. The second pathway involved the reaction of K compounds (such as K_2_O and K_2_CO_3_) with aromatic carbon structures (C–C and C–H) at high temperatures to generate metal K and CO gas.^45^ The number of condensed aromatic rings in M_340_ was greater than that in RC, which can retain more aromatic carbon structures in the coal matrix to react with K compounds at high temperatures; so the CO release of M_340_ is significantly increased at 900 °C. CO_2_ was generated by decarboxylation.^46^ According to FTIR analysis, unstable carboxyl groups are considerably removed during hydromodification; therefore, the CO_2_ release of M_340_ is significantly reduced. Moothi et al.^47^ have reported that CNTs can be prepared from CH_4_ and CO in coal pyrolysis gas by chemical vapor deposition. In this study, lignite inherently released a large amount of CH_4_ and CO during coal pyrolysis (Figure S6), but the CNT content of C-RC is low. In addition, the CH_4_ and CO released during the pyrolysis of modified coal are only ∼13 and ∼15% more than that of lignite, respectively, but the CNT content is greatly improved from ∼2.39 to 9.41%. Obviously, the increase in CH_4_ and CO emissions is limited and cannot cause a significant increase in CNTs content.

#### 2.3.3 Pyrolysis Tar Analysis

**Figure 12:**
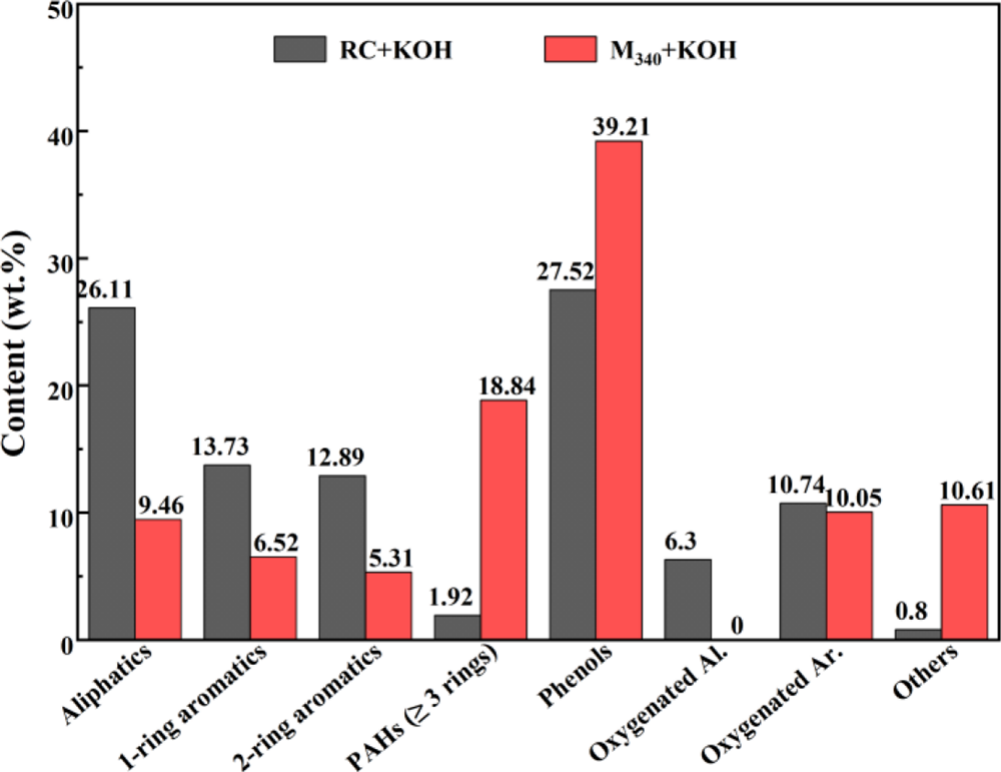
Tar compositions of RC and M_340_.

The tar compositions of RC and M_340_ by gas chromatography/mass spectrometry (GC/MS) analyses (peak area normalization method) is given in Figure 12. The aliphatic content of RC tar was 32.41%, and the content of oxygenated aliphatic hydrocarbons was only 6.3%, mainly because the cracking of oxygenated structures in the tar precursor was promoted by KOH,^46^ which was also confirmed by the high amount of released CO (Figure 11c). The aliphatic content of M_340_ tar was significantly reduced to 9.46% because the aliphatic chain length of M_340_ was shorter and more chains connected to the condensed aromatic rings than those of RC, and the cleavage of short aliphatic chains generated large amounts of gas and polycyclic aromatic hydrocarbons (PAHs). In addition, the oxygenated aliphatic hydrocarbons were almost absent in M_340_ tar, suggesting that the oxygen functional groups in the aliphatic structure are removed or transferred to the aromatic structures during hydromodification, which is confirmed by this experiment. From this analysis, it can be seen that the selectivity of modified coal tar to aliphatic compounds is significantly reduced, confirming that the release of aliphatic compounds during coal pyrolysis slightly affects the growth of CNTs.

The aromatic compound content of RC tar was 66.80%, mainly including phenols, monocyclic aromatic hydrocarbons, bicyclic aromatic hydrocarbons, and oxygenated aromatic hydrocarbons, with contents of 27.52, 13.73, 12.89, and 10.74%, respectively.^48^ Generally, phenols in tar are obtained from the cleavage of aromatic ether bonds.^49^ Peng et al.^50^ have reported that the strong basicity of KOH is beneficial for the conversion of oxygenated aromatics to phenols, leading to the high selectivity for phenols. The content of light aromatics (1- and 2-ring aromatics) was higher, albeit the content of PAHs with ≥3 rings was lower, indicating that the aromatic ring structures in RC is dominated by light aromatic hydrocarbons. The aromatic compound content of M_340_ tar increased significantly to 79.93%, which was 13.13% greater than that of RC tar. The phenol content clearly increased by 11.69%, attributed to the increase in the aryl ether bond and phenolic hydroxyl group content as discussed above. The content of PAHs with ≥3 rings was changed significantly, which was 16.92% greater than that of RC (1.92%). Meanwhile, the light aromatic content of M_340_ tar was reduced by 14.79% than that of RC. The formation of PAHs during coal pyrolysis depends on the structure of the coal. M_340_ possessed more condensed aromatic rings linked by short aliphatic chains than raw coal, and the polycondensation degree of aromatic structure increased, so M_340_ tended to release PAHs with ≥3 rings rather than light aromatics from the coal matrix.^51^ In addition, the presence of KOH promoted the decomposition of PAHs to generate light aromatics;^52^ hence, the pathway of obtaining PAHs from the condensation polymerization of light aromatics is not discussed. From this analysis, the selectivity of the modified coal tar to aromatic compounds is significantly improved. Meanwhile, the CNT yield of modified coal was increased, confirming that the release of aromatic compounds is more conducive to the growth of CNTs, especially PAHs with ≥3 rings and phenols. Moreover, tar molecules were subject to strong diffusional limitations when escaping from the interior of the coal matrix due to their size and reactivity, then the tar molecules or their precursors react sufficiently with active metal K, followed by their conversion into CNTs.^53^

**Figure 13:**
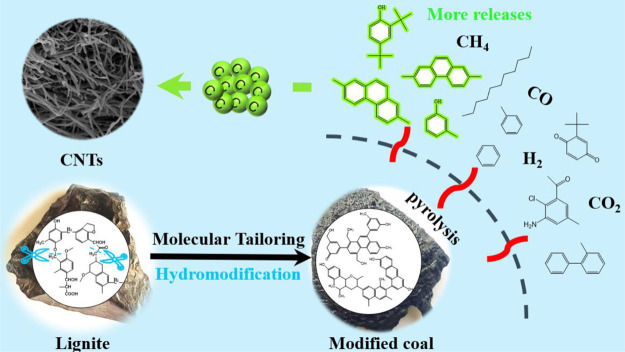
Effect of hydromodification on the CNT formation.

Based on these results, the effect of hydromodification on the CNT formation could be described, as shown in Figure 13. Coal structure has an important influence on CNT growth, and lignite is not an excellent carbon source precursor. Hydromodification can be used as a molecular tailoring strategy to turn the basic aromatic units of lignite into highly active precursors and promote CNT growth. During hydromodification, the lignite structure was tuned to form more aromatic ether bonds and condensed aromatic rings linked by short aliphatic chains, which are the main carbon source precursors. Due to the reconstruction of coal structure, more PAHs with ≥3 rings and phenols were released during pyrolysis of modified coals, which were more conducive to the transformation into the carbon clusters for the CNT growth.^54^ In addition, the CH_4_ and CO released from coal had a limited effect on the growth of CNTs.

## 3 Conclusions

Tuning the lignite structure via hydromodification is an effective method to improve the formation of CNTs in coal-based carbon composites. The formed CNTs were mainly multi-walled CNTs with uniform diameters and the content of CNTs increased gradually with the increase in the modification temperature. As a result, the proportions of loss peaks at 650–670 °C increased from 2.39% of C-RC to 9.41% of C-M_340_. Hydromodification promoted the polycondensation rearrangement of aromatic carbon structures, forming more aromatic ether bonds and condensed aromatic rings linked by short aliphatic chains, which were the main carbon source precursors. During pyrolysis, the coal matrix of modified coal released more aromatic compounds than that released by lignite, especially PAHs with ≥3 rings and phenols, which were more conducive to the transformation into carbon clusters for the CNT growth. In addition, the CH_4_ and CO released from modified coal were about 13 and 15% more than that of lignite, respectively, which had a limited effect on the growth of CNTs during pyrolysis.

## 4 Experimental Section

### 4.1 Materials

Inner Mongolian lignite (RC) and modified lignite (*Mt*, *t* represents the modification temperature) were used as precursors for the preparation of CNT compositions. The hydromodification of coal has been detailed previously.^22^ In brief, 25 g of lignite and 25 g of water were added into a 300-mL autoclave, and then the autoclave reactor was pressurized with 4.5 MPa CO (purity >99.9%) and heated to 280–340 °C for 60 min. After cooling, filtering and drying overnight under vacuum at 80 °C, modified coal was obtained and denoted as Mt., where t represents the hydromodification temperature. The proximate and ultimate analyses of coal samples are presented in Table 4.

**Table 4:** Proximate and Ultimate Analysis of Inner Mongolian Lignite and Modified Coals

samples	proximate analysis (wt %), ad				ultimate analysis (wt %), daf				
	M	V	A	FC	C	H	O^a^	N	S
RC	14.04	39.94	9.22	36.80	66.57	5.97	25.56	1.29	0.61
M_280_	2.18	47.72	11.84	38.26	67.83	5.74	24.31	1.50	0.62
M_310_	2.06	45.17	10.86	41.91	67.94	5.96	23.98	1.82	0.30
M_340_	1.46	40.25	11.93	46.36	73.96	6.41	17.64	1.74	0.25

a By difference.

### 4.2 Methods

#### 4.2.1 Preparation of CNT Composites

**Figure 14:**
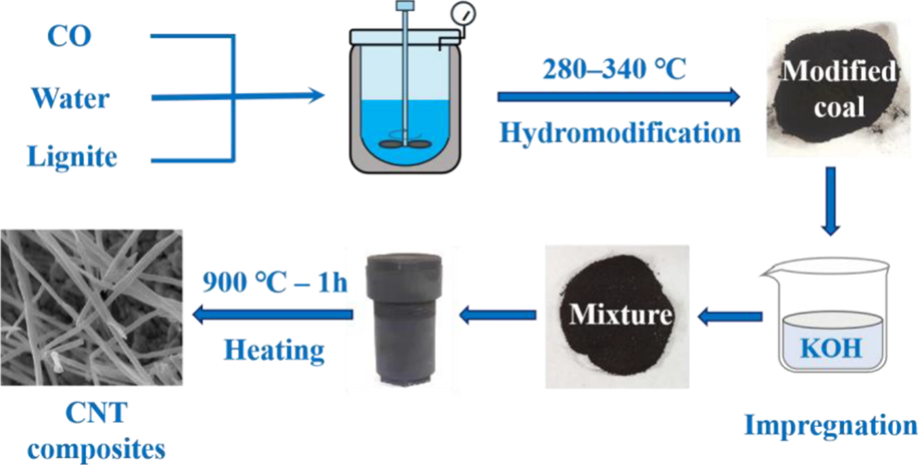
Preparation of CNT composites from modified coal.

Coal samples were loaded with KOH by the impregnation method. The mass ratio of KOH to coal was 1:1, and water was used as the solvent. After stirring for 12 h, the mixture was dried at 105 °C for 12 h, followed by crushing to a grain size of <100 mesh. The prepared mixture was placed into a closed stainless-steel reactor to isolate the air and heated to 900 °C at a heating rate of 10 °C/min. After 60-min reaction, the pyrolysis product was cooled to room temperature and neutralized with 1 mol/L hydrochloric acid to remove the K compounds. Subsequently, the sample was washed with distilled water to neutral pH and dried. The final samples are referred to as C-RC and C-Mt. Figure 14 shows the preparation process of CNT composites form modified coal.

#### 4.2.2 Pyrolysis Experiment in a Fixed Bed Reactor

Figure S8 shows the schematic diagram of a fixed-bed reactor, and the length and outer diameter of the quartz tube were 500 and 30 mm, respectively. Each time, 8 g of the coal-K mixture sample (1: 1) was placed in the reactor, and N_2_ (100 mL/min) is introduced to remove air before experiment. The heating conditions were consistent with those utilized for CNT preparation to reduce the reaction process. Tar and water were collected by cooling, and gas was collected by the drainage method. The yields of tar, water, and solids were calculated by the following formulas (1–3), and the gas yield was obtained by difference subtraction.
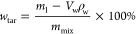
1
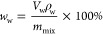
2
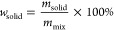
3where *w*_tar_ is the yield of coal pyrolysis tar, %; *w*_w_ is the yield of pyrolysis water, %; *w*_solid_ is the yield of pyrolysis char and K compounds, %; *m*_l_ is the mass of liquid from coal pyrolysis, g; *m*_mix_ is the mass of coal-K mixture, g; *V*_w_ is the volume of water in the liquid, ml; ρ_w_ is the density of water at 298 K and 0.1 MPa, g/cm^3^; and *m*_solid_ is the mass of pyrolysis char and K compounds, g.

The gas was analyzed by gas chromatography (GC 9890A, China). The components of tar were analyzed by GC/MS (FOCUSTM DSQII, USA). The gas chromatography column is DB-5MS (30 m × 0.25 mm × 0.25 μm), 1 mL/min helium gas is used as carrier gas, and the heating program of chromatographic column is set as follows: 40 °C for 4 min, raise to 70 °C at 3 °C/min for 2 min, then raise to 200 °C at 10 °C/min for 3 min, and then raise to 300 °C at 4 °C/min for 5 min. The inlet and transmission line are kept at 300 °C, and the MS power supply is set to 70 eV. The relative content of each compound in tar was calculated by the peak area normalization method according to the NIST database.

### 4.3 Characterization

SEM (TESCAN MIRA4, Czech) was used to observe the surface morphology of the samples, and the acceleration voltage was 20 kV. Nano Measurer software was used to measure the diameter of the CNTs. Importantly, each sample was scanned at different locations to obtain reliable data. 20 points were collected and counted at each location of each sample, and the average outer diameter was obtained by Gaussian fitting.

Transmission electron microscopy (JEM-2100F, Japan) was utilized to characterize the microstructure of CNTs and combined with energy-dispersive X-ray spectroscopy (EDX; METEK I, USA) to determine the elemental composition of the samples by spot scanning.

A laser Raman spectrophotometer (Raman; HR800 HORIBA, Japan) was employed to evaluate the defects of the carbon structure, with an excitation wavelength of 514 nm. The spectra were recorded in the range of 500–3500 cm^–1^.

TGA (NETZSCH STA449F5, Germany) curves were recorded at a heating rate of 10 °C/min to examine the thermal stability of the samples in air.

XRD (LabX-6000, Japan) with Cu Kα radiation (40 kV, 30 mA, λ = 0.15409 nm) was employed to investigate the crystalline structure of coal samples and CNT compositions. The XRD patterns of coal samples were deconvoluted into three Gaussian peaks at ∼18, 25, and 44°, corresponding to the γ, (002), and (100) bands, respectively.^26,27^ The peak center (θ) and full-width at half-maximum were obtained by curve fitting. Structural parameters, including crystallite diameter La, crystallite height Lc, interlayer spacing d_002_, and stacking layer number N were calculated using eqs 4–7, respectively. In addition, the area percentage of the γ bands in the fitting curve was Aγ.
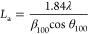
4
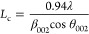
5
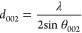
6
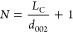
7

An FTIR (Bruker TENSOR II, Germany) spectrometer was utilized to investigate changes of various functional groups of coal samples. FTIR samples were prepared by grinding ∼1.0 mg with 100 mg KBr, and the scanning wavelength ranged from 4000 to 400 cm^–1^. In order to obtain semi-quantitative information of the coal structure, the infrared spectra of coal samples are peak-fitted to obtain absorption peak areas of different functional groups. Infrared parameters of the coal structure are further introduced according to eqs 8–12. *A_x_* represents the peak area at *x* cm^–1^, and these parameters are briefly introduced as follows. C–O/C=O is the content ratio of the C–O bond and C=O bond;^28^ (C–H)_ar_ is the content of the substituted aromatic rings;^29^ C_al_ is the aliphatic hydrocarbon content;^26^ H_ar_/H_al_ is the content ratio of aromatic hydrogen to aliphatic hydrogen;^28^ the higher the CH_2_/CH_3_ content, the longer the aliphatic side chain, and the branching degree is smaller.^1^
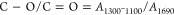
8
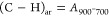
9
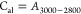
10
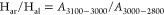
11
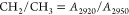
12

## Supporting Information Available

Material characterization and experimental description (PDF).
