# Supplementary material for: Tuning Lignite Structure via Hydromodification To Promote the Formation of Coal-Based CNTs: Exploration for the Carbon Source of CNTs
Source: ACS Omega. 2023 Jul 13;8(29):25938–50. doi: 10.1021/acsomega.3c01736 (PMC10373181; doi:10.1021/acsomega.3c01736)
Supplement: Supplementary file 1 — ao3c01736_si_001.pdf [file ao3c01736_si_001.pdf]

Tuning lignite structure via hydromodification to promote the formation of coal-based CNTs: Exploration for the carbon source of CNTs

Qingxiang Guo [a, b], Yuqiong Zhao [a, b, c, *], Yaning Lei [a, b], Guoqiang Li [a, b], Yajun He [a, b], Guojie Zhang [a, b], Yongfa Zhang [a, b], Kunjie Li [c]

[a] *State Key Laboratory of Clean and Efficient Coal Utilization, Taiyuan University of Technology, Taiyuan 030024, Shanxi, China*

[b] *Key Laboratory of Coal Science and Technology, Ministry of Education, Taiyuan University of Technology, Taiyuan 030024, Shanxi, China*

[c] *Shanxi Huaxin Gas Energy Research Institute Co., Ltd., Taiyuan 030032, Shanxi, China*

Contents:

1. Supporting description of experimental

2. Supporting Tables

Table S1. Absorption peak areas of coal samples by peak separated and calculated.

3. Supporting Figures

Figure. S1 SEM images of C-M_{340} after oxidation at 550 ℃ for different times: (a) 10 min, (b) 30 min.

Figure. S2 XRD patterns of C-M_{340} after oxidation at 550 ℃ for different times.

Figure. S3. SEM images of C-M_{350}.

Figure. S4. TG-DTG analysis of C-M_{350}.

Figure. S5. Curve-fitting of FTIR spectra of RC, M_{280} and M_{310}.

---

*Corresponding author.

E-mail address: zhaoyuqiong@tyut.edu.cn (Yuqiong Zhao)

Figure. S6. Gas yield of RC and M_{340}.

Figure. S7. GC/MS chromatograms of the tar in RC and M_{340}.

Figure. S8. Schematic diagram of a fixed-bed reactor

1. Supporting description of experimental

Oxidative separation of carbon composites

The carbon nanotube composite materials were heated to 500–580 ℃ in a tube furnace for oxidation treatment, and the temperature was programmed at a heating rate of 10 ℃/min in air atmosphere (100 mL/min).

2. Supporting tables

Table. S1.

Absorption peak areas of coal samples by peak separated and calculated.

Peak/cm^{-1}	RC	M_{280}	M_{310}	M_{340}
750	0.24	0.23	0.35	0.36
790	0.24	0.37	0.27	0.46
818	0.09	0.28	0.40	0.25
870	0.12	0.01	0.01	0.01
1100	0.35	0.60	0.55	0.42
1215	1.26	1.36	1.74	1.75
1296	1.74	2.29	2.44	2.36
1375	1.22	1.48	2.52	1.85
1450	3.93	5.14	5.50	5.32
1604	8.06	10.56	11.72	11.76
1700	1.04	1.01	0.93	0.75
2850	1.91	1.53	1.64	1.86
2890	1.08	0.55	1.05	0.68
2920	2.67	2.28	2.17	2.21
2950	0.63	0.55	0.72	0.87
3060	0.48	0.43	0.60	0.56
3135	0.56	0.43	0.57	0.57
3200	1.16	1.88	1.25	1.57
3290	1.29	2.12	2.51	2.11
3380	1.14	0.72	2.05	2.06
3450	0.36	0.61	1.52	1.20
3500	0.36	0.28	0.58	0.97

3. Supporting Figures

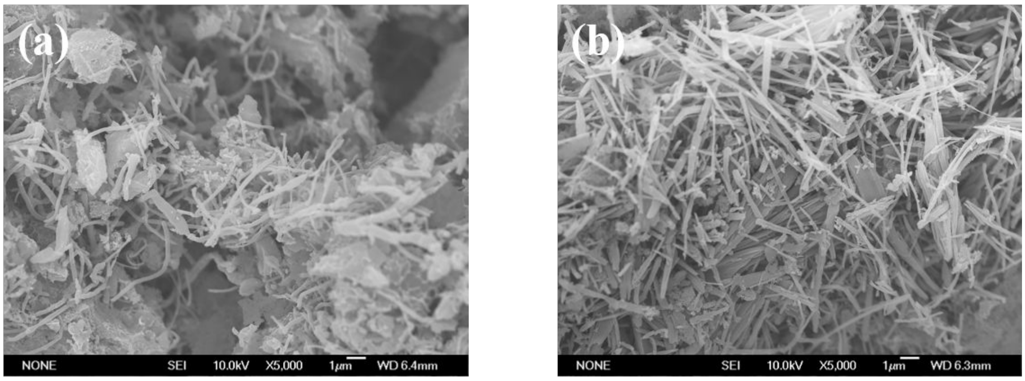

Figure. S1. SEM images of C-M_{340} after oxidation at 550 °C for different times:

(a) 10 min, (b) 30 min.

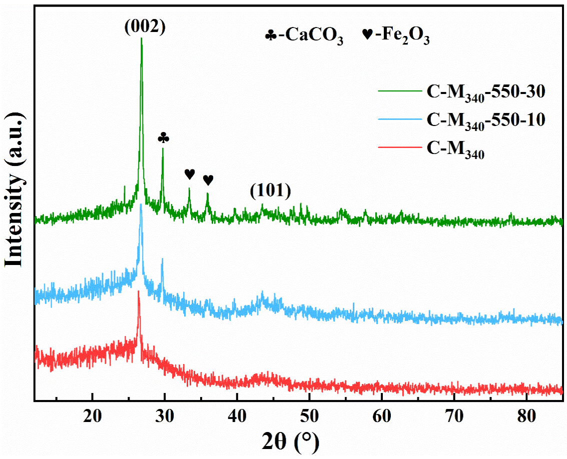

Figure. S2. XRD patterns of C-M_{340} after oxidation at 550 °C for different times.

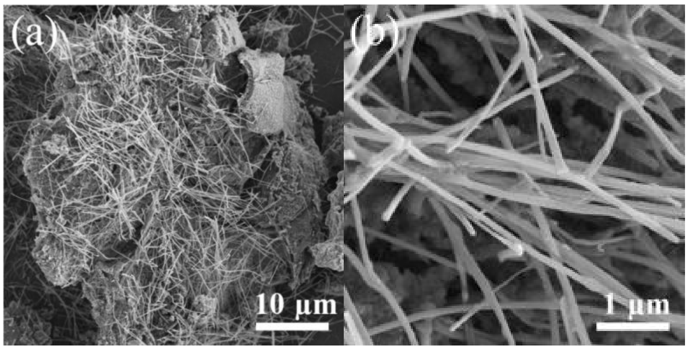

Figure. S3. SEM images of C-M_{350}: (a) 10 μm, (b) 1 μm.

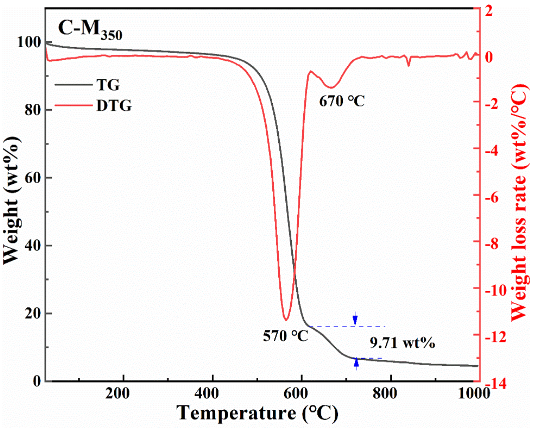

Figure. S4. TG-DTG analysis of C-M_{350}.

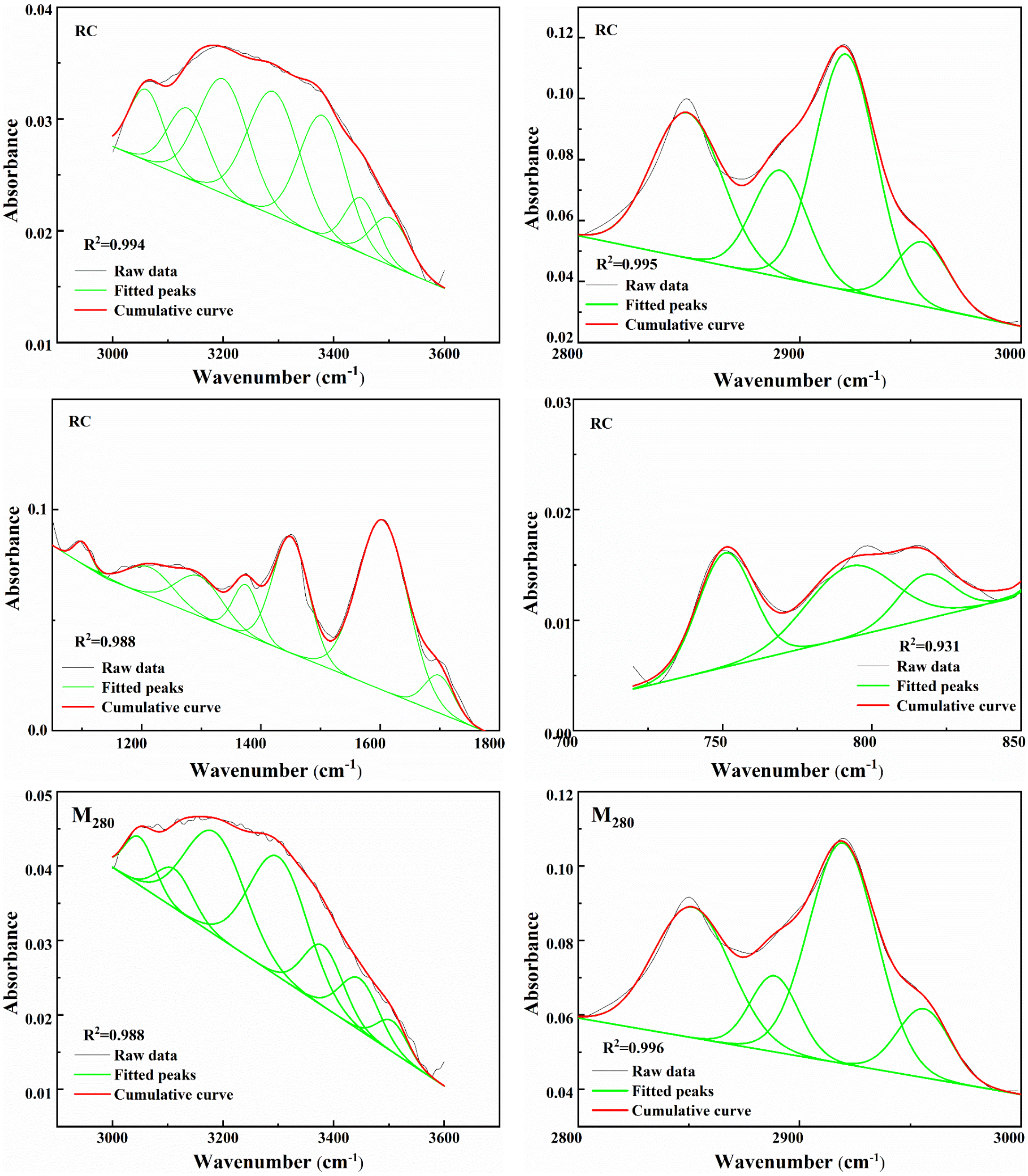

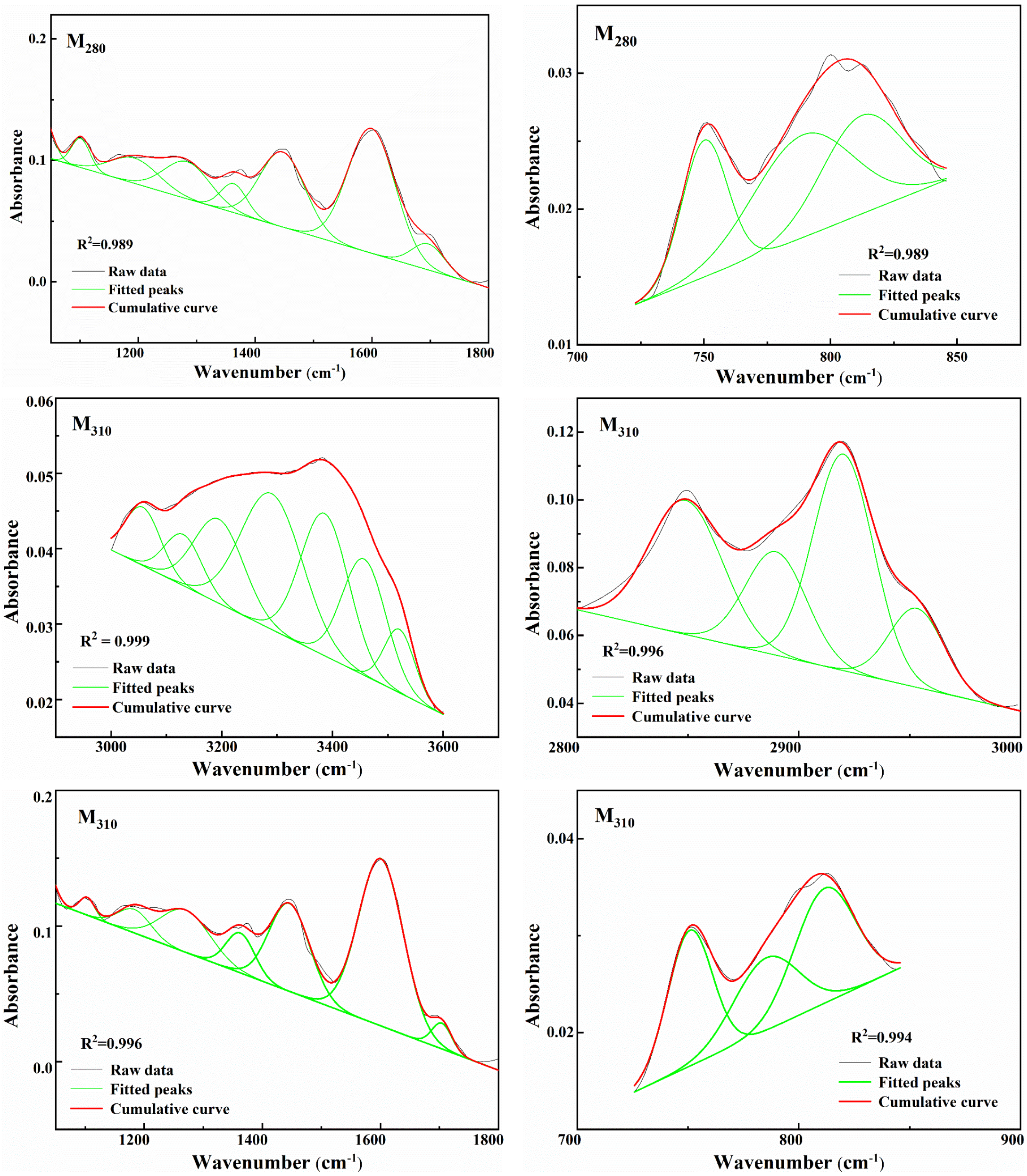

Figure. S5. Curve-fitting of FTIR spectra of RC, M_{280} and M_{310}.

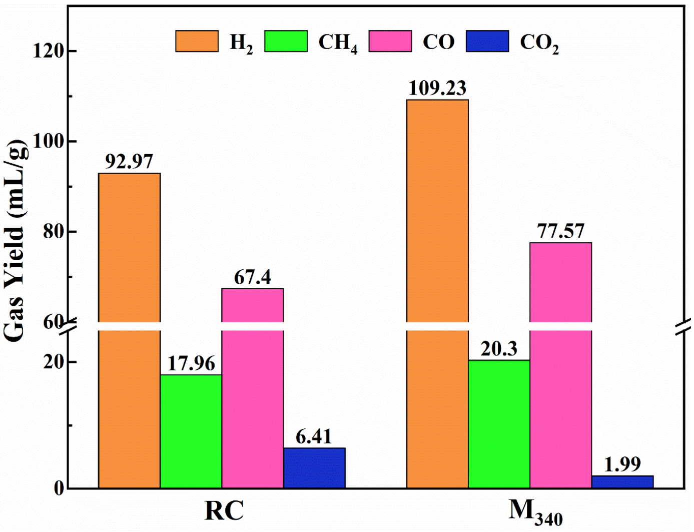

Figure. S6. Gas yield of RC and M_{340}.

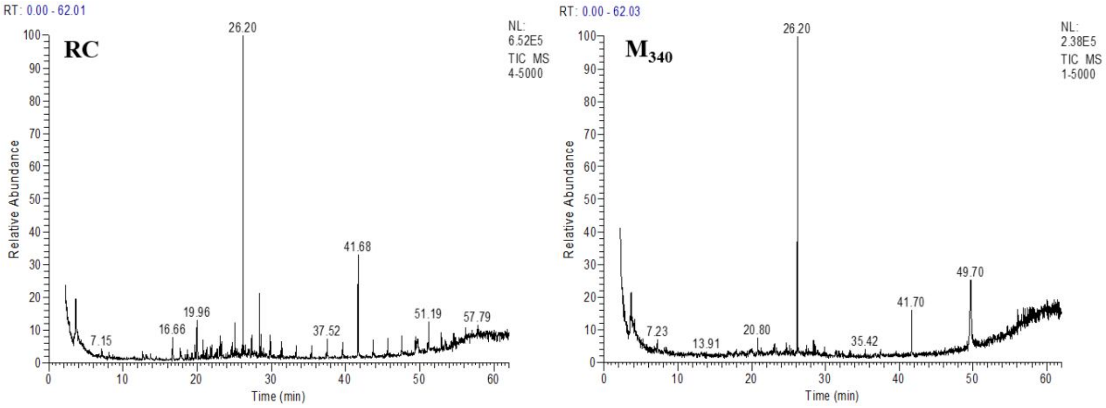

Figure. S7. GC/MS chromatograms of the tar in RC and M_{340}.

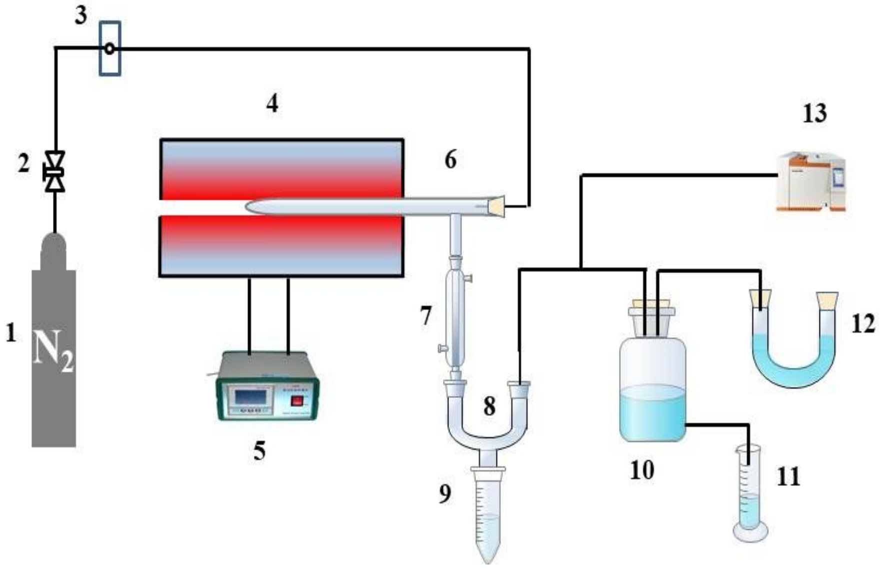

Figure. S8. Schematic diagram of a fixed-bed reactor

1-N_2, 2-valve, 3-rotameter, 4-pyrolysis furnace, 5-programmed temperature controller, 6-quartz

tube, 7-condenser tube, 9-tar collection tube, 10-gas container, 12-U tube differential pressure gauge, 13-gas chromatograph.
